# Associations between nitric oxide synthase 3 gene polymorphisms and preeclampsia risk: a meta-analysis

**DOI:** 10.1038/srep23407

**Published:** 2016-03-21

**Authors:** Fangfang Zeng, Sui Zhu, Martin Chi-Sang Wong, Zuyao Yang, Jinling Tang, Keshen Li, Xuefen Su

**Affiliations:** 1 School of Public Health and Primary Care, Faculty of Medicine, The Chinese University of Hong Kong, Hong Kong, China; 2 CUHK Shenzhen Research Institute, Shenzhen 518057, China; 3 Department of Epidemiology and Biostatistics, West China School of Public Health, Sichuan University, Sichuan 610044, China; 4 Stroke Center, Neurology & Neurosurgery Division, The Clinical Medicine Research Institute & The First Affiliated Hospital, Jinan University, Guangzhou 510630, China

## Abstract

Previous studies have examined the role of three NOS3 gene polymorphisms [G894T, T-786C, and the variable number of tandem repeats 4b/a (VNTR 4b/a)] in the susceptibility to preeclampsia with inconclusive findings. We therefore conducted an updated meta-analysis by including more studies. The most appropriate genetic model was chosen for each polymorphism by using a well-established method. Pooled results indicated that, compared with the GT + GG genotype, the TT genotype of G894T was associated with an increased risk of preeclampsia (odds ratio (OR) = 1.46; 95% confidence interval (CI) = 1.21–1.77, P < 0.001; I^2^ = 40.2%). The CC genotype of T-786C was also associated with a higher risk of preeclampsia (OR = 1.30; 95% CI = 1.07–1.58, P = 0.034; I^2^ = 46.9%) than the CT + TT genotype. No association was found for VNTR 4b/a. Stratified analysis indicated that the increased risk was evident for high-quality studies both for G894T and T-786C, and for studies conducted among Caucasians and Africans for T-786C. However, the increased risk for T-786C among Africans needs further confirmation due to the high probability of false-positive reports. Our results suggested that G894T and T-786C polymorphisms, but not VNTR 4b/a, were associated with an increased risk of preeclampsia.

Preeclampsia is a clinical syndrome characterized by new-onset of hypertension and proteinuria after 20 weeks of gestation^1^. It afflicts 3–5% of pregnancies and is a major cause of maternal and prenatal morbidity and mortality worldwide^2^,^3^. Essential in the pathogenesis of preeclampsia is endothelial dysfunction due to impaired trophoblast invasion and spiral artery remodeling, resulting in abnormal implantation and placental hypo-perfusion^3^. Although some dietary, environmental, and genetic factors of preeclampsia have been identified, its mechanism is still not well understood; therefore, its prevention remains a challenge.

As a potent vasodilator, circulating nitric oxide (NO) plays a crucial role in endothelial function regulation, blood pressure control, and cardiovascular homeostasis, and NO is essential for a predisposition to preeclampsia^4^. NO has been shown *in vitro* and *in vivo* to modulate placental circulations, and the inhibition of NO production has caused preeclampsia-like syndromes in pregnant rats^5^. Endothelial nitric oxide synthase (eNOS) is an enzyme which synthesizes NO constitutively via catalyzing the conversion of l-arginine to l-citrulline^4^. Because endothelial NO availability is largely regulated by its synthesis by eNOS, the gene that encodes eNOS, NOS3, is considered as a candidate gene for preeclampsia^6^.

The NOS3 gene is located on chromosome 7q35–36, with a length of 4.4  kb^7^. The gene comprises 26 exons that encode an mRNA of 4,052 nucleotides^7^. Because the genomic sequence of NOS3 is highly polymorphic, it was of interest to explore which variant(s) in NOS3 might have a functional potential to affect the bioavailability of eNOS and, thus, affect the risk of preeclampsia^7^. Three NOS3 polymorphisms have been extensively studied: G894T (a guanine/thymine substitution at position 894 on exon 7 leading to a change from glutamate to aspartate at position 298; rs1799983)^8^; T-786C mutation (a thymine/cytosine substitution at position 786 in the 5’-flanking region of promoter; rs2070744)^9^; and a variable number of tandem repeats (VNTR) 4b/a polymorphism [the a* -deletion allele with 27 bp VNTR in intron 4]^10^.

In 2013, two meta-analyses on the associations between these three polymorphisms and preeclampsia risk were published but with inconsistent results^11^,^12^. Since the publication of these meta-analyses, eleven new studies have been published^13^,^14^,^15^,^16^,^17^,^18^,^19^,^20^,^21^,^22^,^23^. We have therefore performed an updated systematic review and meta-analysis, adding the recently published studies to further clarify the role of these three SNPs in susceptibility to preeclampsia and to address the limitations of the previous meta-analyses by using more sophisticated methods.

## Results

### Study characteristics

**Figure 1:**
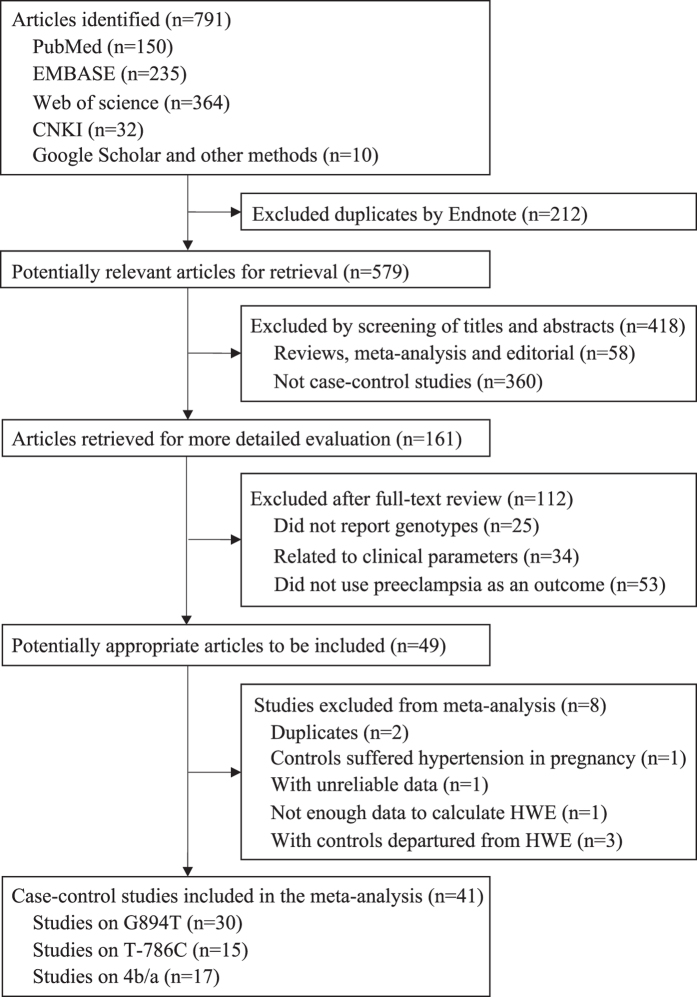
Flow chart of study selection in the meta-analysis.

A total of 791 articles were retrieved by a literature search (Fig. 1). Of the publications that were considered to be possibly relevant for the analysis, the following were excluded: two duplicate publications^24^,^25^, one study^26^ that included controls with hypertension in pregnancy, one study with unreliable data^27^, three studies^28^,^29^,^30^ with controls not in the Hardy-Weinberg equilibrium (HWE), and one study with insufficient data to calculate HWE^31^. Finally, 41case-control studies, including 5,211 cases and 8,779 controls, were used to evaluate the associations of NOS3 polymorphisms (G894T, T-786C, and VNTR 4b/a) with the risk for preeclampsia (Table 1). Thirty articles (3,503 cases and 6,843 controls) were appropriate for combined analysis for G894T, 15 studies (2,232 cases and 3,068 controls) for T-786C, and 17 studies (2,091 cases and 2,638 controls) for VNTR 4b/a.

Table 1 shows the characteristics of included studies. Total sample sizes ranged from 77 to 2,114 (median 255). Twenty-four studies were conducted among Caucasians, 10 among Asians, two among Africans, and the remaining five among mixed Caucasians and Africans. The mean age at study was 27.7 years for the cases and 27.6 years for the controls, and the mean GAD were 35.7 weeks for cases and 39.2 weeks were controls. The quality scores for all of the included studies ranged from five to 14 with a median of eight. Controls in all of the included studies were population-based and were in HWE (Supplementary Table 1).

### Quantitative synthesis

**Figure 2:**
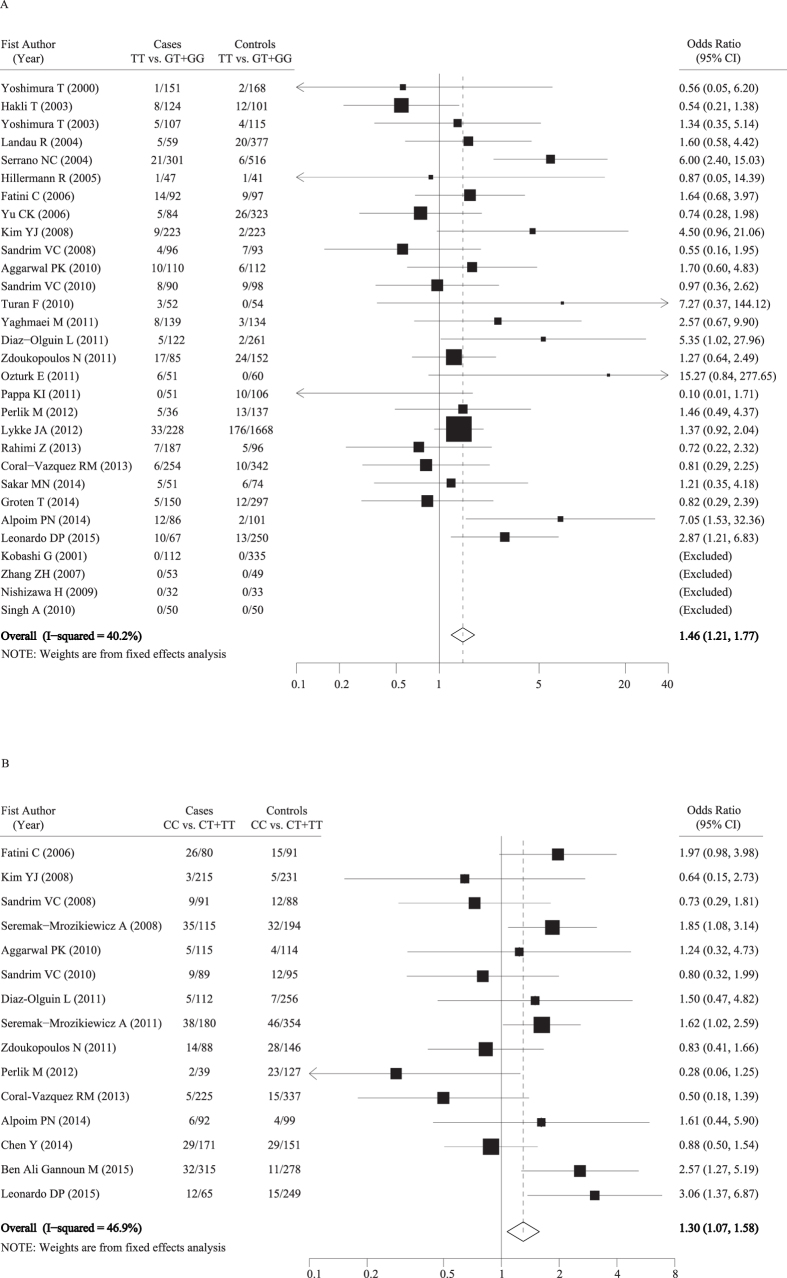
Forest plots of associations between nitric oxide synthase 3 polymorphisms and the risk of preeclampsia (A: TT vs. GT + GG for G894T polymorphism; B: CC vs. CT + TT for T-786C polymorphism).

For SNP G894T, OR1 (GG vs. TT) was 1.48 (P < 0.001); OR2 (GT vs. TT) was 1.11 (P = 0.037); and OR3 (GG vs. GT) was 1.44 (P < 0.001), respectively, suggesting that there is a strong recessive association (TT vs. GT + GG) for the putative susceptibility allele T with preeclampsia. The pooled OR and 95% CI for the association between G894T polymorphism and preeclampsia risk was 1.46 (1.21, 1.77) (P < 0.001) with moderate between-study heterogeneity (I^2^ = 40.2%; Cochran Q = 41.83) (Table 2, Fig. 2). Stratified analysis by the quality scores of included studies revealed a statistically significantly increased risk of preeclampsia only in high-quality studies with scores of 9 to 15 (OR = 1.59, 95% CI = 1.27–2.00; P < 0.001).

For SNPT-786C, OR1 (TT vs. CC) was 1.36 (P = 0.003); OR2 (CT vs. CC) was 1.06 (P = 0.392); and OR3 (TT vs. CT) was 1.26 (P = 0.029), respectively, also suggesting that there is a recessive association (CC vs. CT + TT) of the putative susceptibility allele C with preeclampsia. The overall fixed OR was significant (OR = 1.30; 95% CI = 1.07–1.58, P = 0.008), with moderate between-study heterogeneity (I^2^ = 46.9%; Cochran Q = 26.32). In the stratified analysis by ethnicity, the pooled estimate showed a significantly increased risk in the studies conducted among Caucasians (OR = 1.41; 95% CI = 1.11–1.79, P = 0.005) and among Africans (OR = 2.44; 95% CI = 1.26–4.71, P = 0.008). Similar to SNP G894T, when stratified by quality scores, an increased risk was observed only in high-quality studies with scores of 9 to 15 (OR = 1.47; 95% CI = 1.14–1.89, P = 0.003) (Table 2, Fig. 2).

For the VNTR 4b/a, OR1 (bb vs. aa) was 1.24(P = 0.193); OR2 (ab vs. aa) was 1.00 (P = 0.975); and OR3 (bb vs. ab) was1.07 (P = 0.690), respectively, suggesting that there is no association between the VNTR 4b/a polymorphism and preeclampsia risk. No more models were fitted for VNTR 4b/a.

### Sensitivity analysis and diagnosis of bias

The sensitivity analyses indicated that no single study could change the pooled ORs obviously for both G894Tand T-786C (Table 2). However, after excluding one study by Serrano *et al.*^32^ or Hakli *et al.*^33^ the heterogeneity for G894T was significantly reduced, with I^2^decreasing from 40.2% to 36.0% or 24.9%.

**Figure 3:**
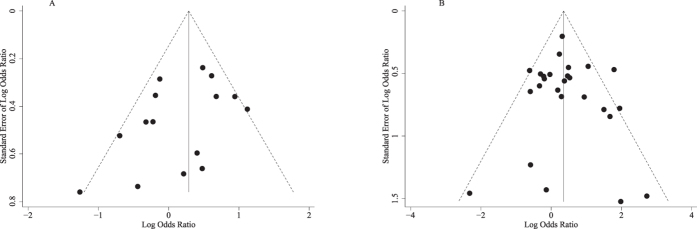
Funnel plots of associations between nitric oxide synthase 3 polymorphisms and the risk of preeclampsia (A: TT vs. GT + GG for G894T polymorphism; B: CC vs. CT + TT for T-786C polymorphism).

Both Egger’s and Begg’s test revealed no significant publication bias, and the P values were 0.634 and 0.508 for G894T, and 0.141and 0.276 for T-786C. The funnel plots also indicated no evidence of publication bias both for G894T and T-786C (Fig. 3).

The false-positive report probability (FPRP) values for significant findings at different prior probability levels are shown in Table 3. For a prior probability of 0.1, assuming that the OR for a specific genotype was 0.67/1.50 (protection/risk), with a statistical power of 0.710, the FPRP value was 0.009 for G894T with an increased risk of preeclampsia, under the recessive genetic model. A positive association was also observed in the high-quality studies for G894T. Similarly, T-786C was also significantly associated with a preeclampsia risk under the recessive genetic model, with the statistical power of 0.590 and the FPRP value of 0.011, and positive associations were found in the subgroup of Caucasians and high-quality studies. However, we did not find an association between T-786C and preeclampsia risk in the subgroup of Africans due to limited statistical power.

## Discussion

In this meta-analysis, we examined the associations between the three NOS3 gene polymorphisms (G894T, T-786C, and VNTR 4b/a) and the preeclampsia risk. Our results provide evidence of a significantly increased risk for preeclampsia for G894T and T-786C polymorphisms, under the recessive genetic model. No association was found between VNTR 4b/a and preeclampsia risk. In addition, stratification analysis showed that the association was more evident for high-quality studies both for G894T and T-786C, and among mixed Caucasian and African populations for T-786C. However, the results for Africans for T-786C need further confirmation due to the high probability of false-positive reports.

Recently, two meta-analyses were published focusing on NOS3 G894T, T-786C, and VNTR 4b/a polymorphisms and the preeclampsia risk^11^,^12^. The meta-analysis by Qi *et al.*^12^ included 26 studies of 2,863 cases and 5,726 controls for G894T, 13 studies of 1,799 cases and 5,853 controls for T-786C, and 15 studies of 1,666 cases and 4,331 controls for T-786C. In the meta-analysis by Qi *et al.*^12^, an increased risk of preeclampsia was observed for G894T in the recessive model (OR = 1.43; 95% CI = 1.13–1.82, P = 0.003) with significant between-study heterogeneity (I^2^ = 50.0%), but no association was observed for T-786C and VNTR 4b/a. Another meta-analysis by Dai *et al.*^11^ included 22 studies of 2,265 cases and 3,709 controls for G894T, 11 studies of 1,538 cases and 2,085 controls for T-786C, and 11 studies of 1,266 cases and 1,643 controls for VNTR 4b/a polymorphisms. The results found that T-786C polymorphism was associated with an increased preeclampsia risk in the dominant model (OR = 1.17, 95% CI = 1.02 − 1.35; I^2^ = 44.2%) and that VNTR 4b/a was associated with a high risk in the recessive model (OR = 1.46, 95% CI = 1.01–2.10; I^2^ = 30.6%). However, no association was observed for G894T (OR = 1.25, 95% CI = 0.96–1.63; I^2^ = 47.0%).

Our study, with the largest sample size, found a significantly increased risk of preeclampsia for G894T and T-786C polymorphisms, but not for VNTR 4b/a, which were partially consistent with the previous meta-analyses. In addition to the larger number of studies included leading to an increased statistical power, the following reasons could also explain the discrepancy. Firstly, the two previous meta-analyses did not exclude studies with controls not in HWE from the pooled analysis, and this may introduce potential selection bias in their findings. Not excluding studies with HWE-violation may result in the overestimation of the statistical significance of some postulated gene-disease associations, and it also seemed to modestly increase the between-study heterogeneity in some instances, thus distorting the findings^34^. In the current meta-analysis, seven studies for G894T^14^,^17^,^28^,^29^,^30^,^31^,^35^, two studies for T-786C^32^,^36^, and four studies for VNTR 4b/a^14^,^18^,^21^,^37^ deviated from HWE. After adding these studies into the pooled analyses, the overall risk and between-study heterogeneity were not significantly changed except for a significant reduction of heterogeneity for G894T (from 52.5% to 38.8%). However, no significant change was found for T-786C. Secondly, some extracted data in the previous meta-analysis (*e.g*., revealed in Dai *et al.*^11^ Table 1), were not accurate. In addition, studies with unreliable datasets (Tempfer *et al.*^27^), with controls having hypertension in pregnancy (Sandrim *et al.*^26^), and with duplicate publication^24^,^25^ were included in the previous meta-analyses^11^,^27^, which may also explain the different results found between our study and the previous meta-analyses.

The evidence that NOS3 G894T and T-786C polymorphisms increase the risk of preeclampsia is biologically plausible. The NOS3 gene is responsible for encoding eNOS, a critical enzyme synthesizing NO through the conversion of L-arginine to L-citrulline in the vascular endothelium, by using molecular oxygen^4^. Circulating NO, the biologically active free radical, plays critical roles in vascular homeostasis^38^. Endogenously synthesized NO promotes tissue perfusion by the relaxation of vascular smooth muscle. It may also protect against foam cell formation and media hypertrophy^39^. A reduced production of NO in women with preeclampsia compared with normal pregnant women was observed^40^. The chronic inhibition of NO synthesis in pregnant rats led to preeclampsia-like syndromes, such as sustained hypertension, proteinuria, thrombocytopenia, and intrauterine growth retardation^5^. NOS3 G894T polymorphism is located in exon 7 leading to a change from glutamate to aspartate at position 298. This variation was found to be susceptible to cleavage by proteases in endothelial cells and vascular tissue, thus leading to reduced vascular NO synthesis^41^. G894T has been suggested to reduce eNOS mRNA expression, its activity and NO levels among patients with preeclampsia compared with healthy controls^29^,^31^. The T-786C SNP is located in the 5′-flanking region of promoter^9^. Carriers with the C allele had a significantly reduced luciferase reporter activity, and decreased eNOS transcription and endothelial production of NO^9^, thereby leading to an increased preeclampsia risk.

In the stratified analysis by ethnicity for T-786C, a significantly increased risk of preeclampsia was found in studies conducted among Caucasians and Africans. The significant finding for Caucasians was probably due to the larger sample size in this subgroup. For Africans, the calculation of the FPRP values suggested that the possibility of false-positive reports could not be completely ruled out, and this still needs further confirmation. Stratified analyses also found significant results for high-quality studies both for G894T and T-786C polymorphisms, which may suggest an underestimation of the overall effects for these two polymorphisms because of suboptimal studies.

One strength of our meta-analysis is a thorough literature search and review to identify as many studies as possible which are relevant to this topic. To the best of our knowledge, this meta-analysis included the largest number of original investigations in this area, thereby giving it the most sufficient study power. In addition, efforts were made to assess publication biases through Egger’s and Begg’s regression asymmetry test and funnel plot, and to pinpoint the potential sources of heterogeneity via stratified and sensitivity analyses. In addition, we used an *a priori* method to determine the most appropriate genetic model and calculated the FPRP to rule out the potential false-positive reports.

Several limitations of the present study also need to be taken into consideration. Firstly, moderate heterogeneity was found for G894T (40.2%) and T-786C (46.9%). For G894T, after excluding one of the studies^32^ from the overall analysis, the heterogeneity reduced to 24.9%, but with the result not being significantly changed. However, both subgroup and sensitivity analysis did not find out the source of the heterogeneity for T-786C, and this needs further investigation. Secondly, some studies included a category of mixed ethnicity for their study population. These populations could not be included in the ethnicity-specific analysis. Thirdly, some subgroup analysis, *e.g*., the pooled sample sizes for the subgroup analyses among Asians and Africans for both G894T and T-786C were relatively small (<1,000 for cases), and this may have attenuated the statistical power. Thus, more studies with larger sample sizes are needed to confirm the associations observed in these subgroup analyses. Finally, due to missing information about disease status (*e.g*., early or late onset; mild or severe disease status), we cannot further explore the associations between NOS3 polymorphisms and preeclampsia risk by disease status, and this may also have influenced the interpretation.

In conclusion, this meta-analysis demonstrates that G894T and T-786C polymorphisms, but not VNTR 4b/a, in the NOS3 gene are associated with an increased risk of preeclampsia. The risk is more evident in high-quality studies both for polymorphisms and in studies conducted among Caucasians for T-786C. The risk for the T-786C polymorphism among Africans needs to be further confirmed. More high-quality studies should be conducted to establish the evidence of the associations between the NO synthase 3 gene polymorphisms and the preeclampsia risk for specific ethnic groups and by disease status.

## Materials and Methods

### Literature search strategies

We followed the standard criteria in the Meta-analysis Of Observational Studies in Epidemiology (MOOSE) guidelines to conduct and report this systematic review^42^. A systematic literature search of PubMed, Embase, Web of Science, and China National Knowledge Infrastructure (CNKI, http://www.cnki.net) up to 24 November 2015 was performed. The following search terms were used: (“pregnancy induced hypertension” or pre-eclampsia or preeclampsia or pre eclampsia) and (“nitric oxide synthase” or eNOS or NOS3) and (polymorphism* or allele* or variant* or mutation* or gene or genetic or genotype). No language restriction was applied; non-English articles were translated if necessary. We also scanned the reference lists of retrieved studies and review articles (including meta-analyses) to identify all of the relevant studies on this topic that might have been missed in database searches. Related articles generated by Google Scholar (http://scholar.google.com) and PubMed were also retrieved.

### Inclusion and exclusion criteria

Two reviewers (ZFF and ZS) identified articles eligible for further review by independently performing an initial screen of identified titles and abstracts independently. Studies were considered for inclusion if they met the following criteria: (1) they were a case–control study (including nested case-control studies); (2) they used preeclampsia as an end point; (3) they included only apparently healthy controls (i.e., people without known hypertension in pregnancy); (4) they included at least one of the three polymorphisms: G894T, T-786C, and VNTR 4b/a; and (5) they provided SNP genotype data and odds ratios (ORs) and corresponding 95% CIs. Studies were excluded if genotype frequency data in the controls demonstrated a departure from HWE. For the studies with overlapping data, only the most relevant articles with the largest dataset were included in the final analysis. Articles were retained when either of the two reviewers believed that they should be retained.

### Data extraction and quality assessment

Data were extracted by using a pilot-tested data extraction form. The following data were extracted: article title, the first author’s name, year of publication, country of the study performed, ethnicity of the study population (Caucasian, Asian, African, etc.), number of cases and controls, mean age and gestational age at delivery (GAD) of cases and controls, inclusion and exclusion criteria of the study population, source of controls, preeclampsia diagnosis criteria, genotyping method, and the numbers of cases and controls with different genotypes. The ORs and corresponding 95% CIs, after controlling for the minimal and the maximal adjusted number of covariates, were also abstracted.

The quality of each investigation was assessed by quality assessment criteria derived from a previously published meta-analysis of molecular association studies^43^. The quality scores of the studies ranged from 0 to 15, with 9 to 15 points indicating a high-quality study and 0 to 8 points indicating a low-quality one. The two reviewers (ZFF and ZS) independently extracted data and assessed the quality of each study, and any discrepancy was resolved through discussion between the two reviewers until a consensus was reached.

### Statistical analysis

The departure of frequencies from expectation under HWE was assessed by chi-square goodness-of-fit tests in controls for each study. The strength of association between the three polymorphisms and preeclampsia risk was assessed by calculating ORs with the corresponding 95% CIs. We used the method proposed by Thakkinstian *et al.*^38^ to define the appropriate genetic model for each polymorphism. Briefly, three ORs were calculated for each polymorphism (G894T: OR1: TT vs. GG, OR2: GT vs. GG, OR3: TT vs. GT; T-786C: OR1: CC vs. TT, OR2: CT vs. TT, OR3: CC vs. CT; and VNTR 4b/a: OR1: aa vs. bb, OR2: ab vs. bb, OR3: aa vs. ab, respectively). Then, the most appropriate genetic model was determined by the above pair-wise differences: If OR1 = OR3 ≠ 1 and OR2 = 1, then a recessive model is suggested; If OR1 = OR2 ≠ 1 and OR3 = 1, then a dominant model is suggested; If OR2 = 1/OR3 ≠ 1 and OR1 = 1, then a complete overdominant model is suggested; If OR1 > OR2 > 1 and OR1 > OR3 > 1 (or OR1 < OR2 < 1 and OR1 < OR3 < 1), then a co-dominant model is suggested.

We used the Chi square-based Q-test to assess between-study heterogeneity. The heterogeneity was also quantified with I^2^ statistics. If no significant heterogeneity was found between the studies, the pooled OR was calculated by using the fixed effects model (the Mantel–Haenszel method)^44^. Otherwise, the random effects model (the DerSimonian and Laird method) was applied^45^. The FPRP was calculated to evaluate the significant findings, as described previously^46^. 0.2 was set as an FPRP threshold and assigned a prior probability of 0.1 to detect an OR of 0.67/1.50 (protective/risk effects) for an association with the genotypes under investigation^47^. An FPRP value <0.2 was considered as a noteworthy finding. We also performed subgroup analysis according to ethnicity and quality scores, respectively. Sensitivity analysis was used to assess the effect of a single study on the summary results^48^. Egger’s and Begg’s regression asymmetry test and funnel plot were used to detect publication bias^39^,^49^. Statistical analysis was performed with Stata Version 11.0 (College Station, TX, USA), and a two-sided P < 0.05 was considered statistically significant.

## Additional Information

**How to cite this article**: Zeng, F. *et al.* Associations between nitric oxide synthase 3 gene polymorphisms and preeclampsia risk: a meta-analysis. *Sci. Rep.* **6**, 23407; doi: 10.1038/srep23407 (2016).

## Supplementary Material

Supplementary Table

## Figures and Tables

**Table 1:** Characteristics of studies included in the meta-analysis.

First author	Year	Country	Ethnicity	Cases			Controls			Study quality score	Genotypes
				N	Age	GAD (weeks)	N	Age	GAD (weeks)		
Aggarwal PK	2010	India	Asian	120	25.7	33.2	118	26.3	35.9	9	G894T, T-786C, VNTR 4b/a
Alpoim PN	2014	Brazil	Caucasian	98	25.5	–	103	24	–	7	G894T, T-786C
Bashford MT	2001	USA	Caucasian	87	25	37.4	53	26	39.9	8	VNTR 4b/a
Ben Ali Gannoun M	2015	Tunisia	African^a^	345	31.4	35.6	289	30.5	38.2	10	T-786C
Benedetto C	2007	Italy	Caucasian	120	30	34	103	30	39	8	VNTR 4b/a
Chen LK	2007	Taibei	Asian	92	30.2	37.6	256	29.7	38.3	10	VNTR 4b/a
Chen Y	2014	China	Asian	220	29.1	36.4	200	27.2	38.1	8	T-786C, VNTR 4b/a
Coral-Vazquez RM	2013	Mexico	Caucasian	230	25.1	–	352	24.6	–	9	G894T, T-786C
Diaz-Olguin L	2011	Mexico	Caucasian	127	22	–	263	21.6	–	9	G894T, T-786C
Fatini C	2006	Italy	Caucasian	106	29	37	106	28	40.5	9	G894T, T-786C, VNTR 4b/a
Groten T	2014	Germany	Mixed^a^	158	–	34.2	312	–	39.3	8	G894T, VNTR 4b/a
Hakli T	2003	Finland	Caucasian	132	28.8	34.7	113	28.7	39.8	8	G894T
Hillermann R	2005	South Africa	African^a^	50	21	30	50	29	39	7	G894T
Kim YJ	2008	Korea	Asian	223	31	35.7	237	31.1	39.1	9	G894T, T-786C
Kobashi G	2001	Japan	Asian	112	29.6	37	335	29.3	39.1	7	G894T
Landau R	2004	USA	Caucasian^a^	64	28	–	397	29	–	10	G894T
Leonardo DP	2015	Brazil	Caucasian	77	26.4	35.2	266	24.5	38.7	10	G894T, T-786C, VNTR 4b/a
Lykke JA	2012	Denmark	Caucasian	263	30.2	35.3	1,851	30.3	39.9	14	G894T
Mozgovaia EV	2001	Russia	Caucasian	122	–	–	73	–	–	5	VNTR 4b/a
Nishizawa H	2009	Japan	Asian	33	30.6	–	44	29.5	–	8	G894T
Ozturk E	2011	Turkey	Caucasian	57	29.05	34.54	60	30.2	36.58	9	G894T, VNTR 4b/a
Pappa KI	2011	Greece	Caucasian	51	26	–	116	27	–	7	G894T
Perlik M	2012	Poland	Caucasian	41	29.46	36.88	150	28.3	39.06	5	G894T, T-786C
Rahimi Z	2013	Iran	Caucasian	179	29.2	–	96	27.5	–	9	VNTR 4b/a
Rahimi Z	2013	Iran	Caucasian	198	29.1	–	101	27.4	–	9	G894T
Sakar MN	2014	Turkey	Caucasian	56	28.39	35.41	80	28.2	39.11	7	G894T
Salimi S	2012	Iran	Caucasian	123	28	36.6	142	26.5	37.9	9	VNTR 4b/a
Sandrim VC	2010	Brazil	Mixed^a^	98	26.4	36	107	24.8	40.9	9	G894T, T-786C, VNTR 4b/a
Sandrim VC	2008	Brazil	Mixed^a^	113	26.4	35.9	110	26	40.7	9	G894T, T-786C, VNTR 4b/a
Seremak-Mrozikiewicz A	2008	Poland	Caucasian	150	28.3	35.4	226	27.9	39.4	6	T-786C
Seremak-Mrozikiewicz A	2011	Poland	Caucasian	218	28.6	35.7	400	27.3	39.2	9	T-786C
Serrano NC	2004	Colombia	Mixed^a^	322	19.2	36.4	522	18.9	39.1	10	G894T, VNTR 4b/a
Singh A	2010	India	Asian	50	–	–	50	–	–	7	G894T
Tempfer CB	2001	USA	Caucasian^a^	66	26	36.7	44	25	39.9	7	VNTR 4b/a
Turan F	2010	Turkey	Caucasian	55	32	–	54	29.6	–	7	G894T
Yaghmaei M	2011	Iran	Caucasian	147	28.1	36.6	137	26.3	38.2	8	G894T
Yoshimura T	2003	Japan	Asian	112	24.5	–	119	24.6	–	8	G894T
Yoshimura T	2000	Japan	Asian	152	30	36	170	30.6	39.4	9	G894T
Yu CK	2006	UK	Mixed^a^	89	–	–	349	–	–	11	G894T
Zdoukopoulos N	2011	Greece	Caucasian	102	30.64	35.41	176	29.6	38.58	8	G894T, T-786C, VNTR 4b/a
Zhang ZH	2007	China	Asian	53	29.2	–	49	28.7	–	5	G894T

GAD: gestational age at delivery; VNTR: variable number of tandem repeats;

^a^Other refers to mixed ethnicity with Caucasians and Africans.

**Table 2:** Total and stratified analysis of nitric oxide synthase 3 polymorphisms and the preeclampsia risk.

Summary	N^a^	Cases/Controls	OR (95%CI)	Cochran Q	I^2^, %	*P* for Z test^b^
**G894T**						
Total	30	3,503/6,843	1.46 (1.21, 1.77)	41.83	40.2	**<0.001**
Ethnicity						
Caucasian	20	2,108/5,020	1.29 (0.90, 1.85)	39.84	52.2	0.173
Asian	8	863/1,099	1.77 (0.88, 3.55)	2.47	0.0	0.108
African	4	219/318	1.81 (0.43, 7.58)	1.49	0.0	0.416
Mixed^c^	2	311/431	2.23 (0.43, 11.55)	5.35	81.2	0.339
Score						
Low	15	1,244/1,866	1.22 (0.87, 1.72)	14.22	29.7	0.240
High	15	2,259/4,977	1.59 (1.27, 2.00)	26.64	47.4	**<0.001**
Sensitivity analysis						
Maximal	29	−/−	1.53 (1.26, 1.86)	31.94	36.0	**<0.001**
Minimal	29	−/−	1.35 (1.11, 1.64)	23.69	24.9	**0.003**

**T-786C**						
Total	15	2,232/3,068	1.30 (1.07, 1.58)	26.32	46.9	**0.008**
Ethnicity						
Caucasian	10	1,227/2,115	1.41 (1.11, 1.79)	19.53	53.5	**0.005**
Asian	3	538/534	0.89 (0.55, 1.45)	0.43	0.0	0.641
African	2	369/312	2.44 (1.26, 4.71)	0.18	0.0	**0.008**
Mixed^c^	1	98/107	0.80 (0.32, 1.99)	0.00	−	0.632
Score						
Low	5	591/833	1.08 (0.79, 1.47)	8.43	52.5	0.636
High	10	1,641/2,235	1.47 (1.14, 1.89)	16.03	43.9	**0.003**
Sensitivity analysis						
Maximal	14	−/−	1.37 (1.12, 1.69)	22.79	45.9	**0.003**
Minimal	14	−/−	1.22 (1.01, 1.49)	20.57	43.0	**0.045**

OR: odds ratio; CI: confidence interval;

^a^Number of studies.

^b^P-value of Z-test for significance.

^c^Mixed ethnicity included both Caucasians and Africans.

**Table 3:** False-positive report probability values for associations between the nitric oxide synthase 3 polymorphisms and the preeclampsia risk.

Genotype	Crude OR (95% CI)	P-value^a^	Statistical power^b^	Prior probability				
				0.25	0.1	0.01	0.001	0.0001
G894T								
Recessive genetic model	1.46 (1.21, 1.77)	**<0.001**	0.710	**0.003**	**0.009**	**0.089**	0.496	0.908
High-quality score	1.59 (1.27, 2.00)	**<0.001**	0.453	**0.005**	**0.014**	**0.133**	0.607	0.939
T-786C								
Recessive genetic model	1.30 (1.07, 1.58)	**0.008**	0.590	**0.004**	**0.011**	**0.105**	0.542	0.922
Caucasian	1.41 (1.11, 1.79)	**0.005**	0.303	**0.007**	**0.020**	**0.186**	0.698	0.958
African	2.44 (1.26, 4.71)	**0.008**	**0.008**	0.204	0.435	0.894	0.988	0.999
High-quality score	1.47 (1.14, 1.89)	**0.003**	0.241	**0.009**	**0.026**	0.224	0.744	0.967

OR: odds ratio; CI: confidence interval;

^a^Chi-square test was used to calculate the genotype and haplotype frequency distributions.

^b^Statistical power was calculated by using the number of observations in the subgroup and the OR and P values in this table.
