# Supplementary material for: Associations between nitric oxide synthase 3 gene polymorphisms and preeclampsia risk: a meta-analysis
Source: Sci Rep. 2016 Mar 21;6:23407. doi: 10.1038/srep23407 (PMC4800677; doi:10.1038/srep23407)
Supplement: Supplementary Table [file srep23407-s1.doc]

[**Associations between nitric oxide synthase 3 gene polymorphisms and preeclampsia risk: a meta-analysis**](http://www.ncbi.nlm.nih.gov/pubmed/26278624)

Fangfang Zeng^1, 2#^, Sui Zhu^3#^, Martin Chi-Sang Wong^1, 2^, Zuyao Yang^1, 2^, Jinling Tang^1, 2^, Keshen Li^4*^, Xuefen Su^1, 2*^

1. School of Public Health and Primary Care, Faculty of Medicine, The Chinese University of Hong Kong, Hong Kong, China;
2. CUHK Shenzhen Research Institute, Shenzhen 518057, China;
3. Department of Epidemiology and Biostatistics, West China School of Public Health, Sichuan University, Sichuan 610044, China;
4. Stroke Center, Neurology & Neurosurgery Division, The Clinical Medicine Research Institute & The First Affiliated Hospital, Jinan University, Guangzhou 510630, China

^#^Fangfang Zeng and Sui Zhu contributed equally to this work and should be considered as co-first authors.

*** Reprint requests and correspondence:**

**Xuefen Su**

School of Public Health and Primary Care, Faculty of Medicine,

The Chinese University of Hong Kong,

Hong Kong, China

Tel.: +85222528740

Fax: +85226453098

E-mail address: xuefensu@cuhk.edu.hk

**Keshen Li**

Stroke Center, Neurology & Neurosurgery Division, The Clinical Medicine Research Institute & The First Affiliated Hospital, Jinan University

Guangzhou 510630, China

Tel.: +86 2038688888

Fax: +86 2038688888

E-mail: likeshen1971@126.com

Supplementary table 1 Nitric oxide synthase 3 genotype distributions among preeclampsia cases and controls of the included studies

| First author | Year | Cases | | |  | Controls | | | MAF | P (HWE) *^a^* |
| --- | --- | --- | --- | --- | --- | --- | --- | --- | --- | --- |
|  |  | 11 | 12 | 22 |  | 11 | 12 | 22 |  |  |
| **G894T** |  |  |  |  |  |  |  |  |  |  |
| Aggarwal PK[^1^](#_ENREF_1) | 2010 | 64 | 46 | 10 |  | 70 | 42 | 6 | 0.229 | 0.926 |
| Alpoim PN[^2^](#_ENREF_2) | 2014 | 50 | 36 | 12 |  | 62 | 39 | 2 | 0.209 | 0.138 |
| Coral-Vazquez RM[^3^](#_ENREF_3) | 2013 | 175 | 79 | 6 |  | 256 | 86 | 10 | 0.151 | 0.400 |
| Diaz-Olguin L[^4^](#_ENREF_4) | 2011 | 94 | 28 | 5 |  | 206 | 55 | 2 | 0.112 | 0.418 |
| Fatini C[^5^](#_ENREF_5) | 2006 | 42 | 50 | 14 |  | 49 | 48 | 9 | 0.311 | 0.564 |
| Groten T[^6^](#_ENREF_6) | 2014 | 114 | 36 | 5 |  | 214 | 83 | 12 | 0.173 | 0.277 |
| Hakli T[^7^](#_ENREF_7) | 2003 | 73 | 51 | 8 |  | 52 | 49 | 12 | 0.323 | 0.928 |
| Hillermann R[^8^](#_ENREF_8) | 2005 | 41 | 6 | 1 |  | 33 | 8 | 1 | 0.119 | 0.551 |
| Kim YJ[^9^](#_ENREF_9) | 2008 | 192 | 31 | 9 |  | 188 | 35 | 2 | 0.087 | 0.794 |
| Kobashi G[^10^](#_ENREF_10) | 2001 | 86 | 26 | 0 |  | 294 | 41 | 0 | 0.061 | 0.233 |
| Landau R[^11^](#_ENREF_11) | 2004 | 43 | 16 | 5 |  | 252 | 125 | 20 | 0.208 | 0.384 |
| Leonardo DP[^12^](#_ENREF_12) | 2015 | 42 | 25 | 10 |  | 149 | 101 | 13 | 0.241 | 0.432 |
| Lykke JA[^13^](#_ENREF_13) | 2012 | 107 | 121 | 33 |  | 888 | 780 | 176 | 0.307 | 0.804 |
| Nishizawa H[^14^](#_ENREF_14) | 2009 | 28 | 4 | 0 |  | 28 | 5 | 0 | 0.076 | 0.638 |
| Ozturk E[^15^](#_ENREF_15) | 2011 | 28 | 23 | 6 |  | 42 | 18 | 0 | 0.150 | 0.172 |
| Pappa KI[^16^](#_ENREF_16) | 2011 | 22 | 29 | 0 |  | 57 | 49 | 10 | 0.297 | 0.908 |
| Perlik M[^17^](#_ENREF_17) | 2012 | 24 | 12 | 5 |  | 73 | 64 | 13 | 0.300 | 0.846 |
| Rahimi Z[^18^](#_ENREF_18) | 2013 | 130 | 57 | 7 |  | 62 | 34 | 5 | 0.218 | 0.903 |
| Sakar MN[^19^](#_ENREF_19) | 2014 | 32 | 19 | 5 |  | 41 | 33 | 6 | 0.281 | 0.856 |
| Sandrim VC[^20^](#_ENREF_20) | 2008 | 55 | 41 | 4 |  | 56 | 37 | 7 | 0.255 | 0.793 |
| Sandrim VC[^21^](#_ENREF_21) | 2010 | 55 | 35 | 8 |  | 52 | 46 | 9 | 0.299 | 0.793 |
| Serrano NC[^22^](#_ENREF_22) | 2004 | 217 | 84 | 21 |  | 403 | 113 | 6 | 0.120 | 0.538 |
| Singh A[^23^](#_ENREF_23) | 2010 | 47 | 3 | 0 |  | 48 | 2 | 0 | 0.020 | 0.885 |
| Turan F[^24^](#_ENREF_24) | 2010 | 29 | 23 | 3 |  | 34 | 20 | 0 | 0.185 | 0.095 |
| Yaghmaei M[^25^](#_ENREF_25) | 2011 | 61 | 78 | 8 |  | 86 | 48 | 3 | 0.197 | 0.210 |
| Yoshimura T[^26^](#_ENREF_26) | 2000 | 122 | 29 | 1 |  | 146 | 22 | 2 | 0.076 | 0.275 |
| Yoshimura T[^27^](#_ENREF_27) | 2003 | 72 | 35 | 5 |  | 77 | 38 | 4 | 0.193 | 0.793 |
| Yu CK[^28^](#_ENREF_28) | 2006 | 59 | 25 | 5 |  | 199 | 124 | 26 | 0.252 | 0.279 |
| Zdoukopoulos N[^29^](#_ENREF_29) | 2011 | 47 | 38 | 17 |  | 68 | 84 | 24 | 0.375 | 0.809 |
| Zhang ZH[^30^](#_ENREF_30) | 2007 | 38 | 15 | 0 |  | 41 | 8 | 0 | 0.082 | 0.534 |
|  |  |  |  |  |  |  |  |  |  |  |
| **T-786C** |  |  |  |  |  |  |  |  |  |  |
| Aggarwal PK [^1^](#_ENREF_1) | 2010 | 80 | 35 | 5 |  | 74 | 40 | 4 | 0.203 | 0.617 |
| Alpoim PN [^2^](#_ENREF_2) | 2014 | 44 | 48 | 6 |  | 59 | 40 | 4 | 0.233 | 0.380 |
| [Ben Ali Gannoun M](http://www.ncbi.nlm.nih.gov.proxy.its.virginia.edu/pubmed/?term=Ben Ali Gannoun M%5BAuthor%5D&cauthor=true&cauthor_uid=26049094)^31^ | 2015 | 160 | 155 | 32 |  | 161 | 117 | 11 | 0.240 | 0.066 |
| Chen Y[^32^](#_ENREF_32) | 2014 | 81 | 90 | 29 |  | 74 | 77 | 29 | 0.375 | 0.241 |
| Coral-Vazquez RM[^3^](#_ENREF_3) | 2013 | 170 | 55 | 5 |  | 240 | 97 | 15 | 0.180 | 0.201 |
| Diaz-Olguin L[^4^](#_ENREF_4) | 2011 | 94 | 28 | 5 |  | 195 | 61 | 7 | 0.143 | 0.404 |
| Fatini C[^5^](#_ENREF_5) | 2006 | 35 | 45 | 26 |  | 45 | 46 | 15 | 0.358 | 0.561 |
| Kim YJ[^9^](#_ENREF_9) | 2008 | 176 | 39 | 3 |  | 185 | 46 | 5 | 0.119 | 0.296 |
| Leonardo DP[^12^](#_ENREF_12) | 2015 | 36 | 29 | 12 |  | 131 | 118 | 15 | 0.280 | 0.080 |
| Perlik M[^17^](#_ENREF_17) | 2012 | 20 | 19 | 2 |  | 59 | 68 | 23 | 0.380 | 0.642 |
| Sandrim VC [^20^](#_ENREF_20) | 2008 | 48 | 43 | 9 |  | 41 | 47 | 12 | 0.355 | 0.792 |
| Sandrim VC[^21^](#_ENREF_21) | 2010 | 46 | 43 | 9 |  | 43 | 52 | 12 | 0.355 | 0.528 |
| Seremak-Mrozikiewicz A [^33^](#_ENREF_33) | 2008 | 42 | 73 | 35 |  | 82 | 112 | 32 | 0.389 | 0.526 |
| Seremak-Mrozikiewicz A[^34^](#_ENREF_34) | 2011 | 64 | 116 | 38 |  | 153 | 201 | 46 | 0.366 | 0.099 |
| Zdoukopoulos N[^29^](#_ENREF_29) | 2011 | 37 | 51 | 14 |  | 62 | 84 | 28 | 0.402 | 0.960 |
|  |  |  |  |  |  |  |  |  |  |  |
| **VNTR 4b/a** |  |  |  |  |  |  |  |  |  |  |
| Aggarwal PK[^1^](#_ENREF_1) | 2010 | 83 | 32 | 5 |  | 74 | 39 | 5 | 0.208 | 0.961 |
| Bashford MT[^35^](#_ENREF_35) | 2001 | 48 | 17 | 5 |  | 40 | 4 | 0 | 0.045 | 0.752 |
| Benedetto C[^36^](#_ENREF_36) | 2007 | 76 | 39 | 5 |  | 69 | 32 | 2 | 0.175 | 0.434 |
| Chen LK[^37^](#_ENREF_37) | 2007 | 83 | 9 | 0 |  | 206 | 50 | 0 | 0.098 | 0.083 |
| Chen Y[^32^](#_ENREF_32) | 2014 | 161 | 37 | 2 |  | 124 | 51 | 5 | 0.169 | 0.929 |
| Fatini C[^5^](#_ENREF_5) | 2006 | 62 | 33 | 11 |  | 72 | 31 | 3 | 0.175 | 0.877 |
| Groten T[^6^](#_ENREF_6) | 2014 | 27 | 48 | 6 |  | 51 | 67 | 15 | 0.365 | 0.315 |
| Leonardo DP[^12^](#_ENREF_12) | 2015 | 52 | 17 | 0 |  | 148 | 78 | 13 | 0.218 | 0.522 |
| Mozgovaia EV[^38^](#_ENREF_38) | 2001 | 73 | 42 | 3 |  | 47 | 24 | 2 | 0.192 | 0.605 |
| Ozturk E[^15^](#_ENREF_15) | 2011 | 37 | 14 | 6 |  | 43 | 16 | 1 | 0.150 | 0.723 |
| Rahimi Z[^39^](#_ENREF_39) | 2013 | 121 | 56 | 2 |  | 69 | 25 | 2 | 0.151 | 0.880 |
| Salimi S[^40^](#_ENREF_40) | 2012 | 80 | 42 | 1 |  | 97 | 41 | 4 | 0.173 | 0.894 |
| Sandrim VC [^20^](#_ENREF_20) | 2008 | 58 | 34 | 8 |  | 54 | 41 | 5 | 0.255 | 0.429 |
| Sandrim VC [^21^](#_ENREF_21) | 2010 | 63 | 33 | 4 |  | 61 | 32 | 7 | 0.230 | 0.334 |
| Serrano NC[^22^](#_ENREF_22) | 2004 | 68 | 33 | 1 |  | 109 | 60 | 7 | 0.210 | 0.724 |
| Tempfer CB[^41^](#_ENREF_41) | 2001 | 253 | 51 | 13 |  | 393 | 103 | 12 | 0.125 | 0.099 |
| Zdoukopoulos N[^29^](#_ENREF_29) | 2011 | 40 | 18 | 8 |  | 40 | 4 | 0 | 0.045 | 0.752 |

HWE: Hardy-Weinberg equilibrium; VNTR: variable number of tandem repeats;

^a^ P value of chi-square test for HWE among controls

**References**

1 Aggarwal, P. K., Jain, V. & Jha, V. Endothelial nitric oxide synthase, angiotensin-converting enzyme and angiotensinogen gene polymorphisms in hypertensive disorders of pregnancy. *Hypertens Res* **33**, 473-477 (2010).

2 Alpoim, P. N. et al*.* Polymorphisms in endothelial nitric oxide synthase gene in early and late severe preeclampsia. *Nitric Oxide* **42**, 19-23 (2014).

3 Coral-Vazquez, R. M. et al*.* Analysis of polymorphisms and haplotypes in genes associated with vascular tone, hypertension and oxidative stress in Mexican-Mestizo women with severe preeclampsia. *Clin Biochem* **46**, 627-632 (2013).

4 Diaz-Olguin, L. et al*.* Endothelial nitric oxide synthase haplotypes are associated with preeclampsia in Maya mestizo women. *Dis Markers* **31**, 83-89 (2011).

5 Fatini, C. et al. Endothelial nitric oxide synthase gene influences the risk of pre-eclampsia, the recurrence of negative pregnancy events, and the maternal-fetal flow. *J Hypertens* **24**, 1823-1829 (2006).

6 Groten, T. et al. eNOSI4 and EPHX1 polymorphisms affect maternal susceptibility to preeclampsia: analysis of five polymorphisms predisposing to cardiovascular disease in 279 Caucasian and 241 African women. *Arch Gynecol Obstet* **289**, 581-593 (2014).

7 Hakli, T. et al. Endothelial nitric oxide synthase polymorphism in preeclampsia. *J Soc Gynecol Investig* **10**, 154-157 (2003).

8 Hillermann, R., Carelse, K. & Gebhardt, G. S. The Glu298Asp variant of the endothelial nitric oxide synthase gene is associated with an increased risk for abruptio placentae in pre-eclampsia. *J Hum Genet* **50**, 415-419 (2005).

9 Kim, Y. J. et al. No association of the genetic polymorphisms of endothelial nitric oxide synthase, dimethylarginine dimethylaminohydrolase, and vascular endothelial growth factor with preeclampsia in Korean populations. *Twin Res Hum Genet* **11**, 77-83 (2008).

10 Kobashi, G. et al. Endothelial nitric oxide synthase gene (NOS3) variant and hypertension in pregnancy. *Am J Med Genet* **103**, 241-244 (2001).

11 Landau, R. et al. No association of the Asp298 variant of the endothelial nitric oxide synthase gene with preeclampsia. *Am J Hypertens* **17**, 391-394 (2004).

12 Leonardo, D. P. et al. Association of Nitric Oxide Synthase and Matrix Metalloprotease Single Nucleotide Polymorphisms with Preeclampsia and Its Complications. *PLoS One* **10**, e0136693 (2015).

13 Lykke, J. A. et al. Vascular associated gene variants in patients with preeclampsia: results from the Danish National Birth Cohort. *Acta Obstet Gynecol Scand* **91**, 1053-1060 (2012).

14 Nishizawa, H. et al. Analysis of nitric oxide metabolism as a placental or maternal factor underlying the etiology of pre-eclampsia. *Gynecol Obstet Invest* **68**, 239-247 (2009).

15 Ozturk, E. et al. Endothelial nitric oxide synthase gene polymorphisms in preeclampsia with or without eclampsia in a Turkish population. *J Obstet Gynaecol Res* **37**, 1778-1783 (2011).

16 Pappa, K. I. et al. Variable effects of maternal and paternal-fetal contribution to the risk for preeclampsia combining GSTP1, eNOS, and LPL gene polymorphisms. *J Matern Fetal Neonatal Med* **24**, 628-635 (2011).

17 Perlik, M. et al. Genetic variants of endothelial nitric synthase in gestational hypertension and preeclampsia. *Ginekol Pol* **83**, 652-659 (2012).

18 Rahimi, Z., Malek-Khosravi, S., Jalilvand, F. & Parsian, A. MTHFR C677T and eNOS G894T variants in preeclamptic women: Contribution to lipid peroxidation and oxidative stress. *Clin Biochem* **46**, 143-147 (2013).

19 Sakar, M. N. et al. Association of endothelial nitric oxide synthase gene G894T polymorphism and serum nitric oxide levels in patients with preeclampsia and gestational hypertension. *J Matern Fetal Neonatal Med* **28**, 1907-1911 (2015).

20 Sandrim, V. C. et al. eNOS haplotypes associated with gestational hypertension or preeclampsia. *Pharmacogenomics* **9**, 1467-1473 (2008).

21 Sandrim, V. C. et al. Effects of eNOS polymorphisms on nitric oxide formation in healthy pregnancy and in pre-eclampsia. *Mol Hum Reprod* **16**, 506-510 (2010).

22 Serrano, N. C. et al. Endothelial NO synthase genotype and risk of preeclampsia: a multicenter case-control study. *Hypertension* **44**, 702-707 (2004).

23 Singh, A., Sharma, D., Raghunandan, C. & Bhattacharjee, J. Role of inflammatory cytokines and eNOS gene polymorphism in pathophysiology of pre-eclampsia. *Am J Reprod Immunol* **63**, 244-251 (2010).

24 Turan, F., Ilhan, N., Kaman, D., Ates, K. & Kafkasli, A. Glu298Asp polymorphism of the endothelial nitric oxide synthase gene and plasma concentrations of asymmetric dimethylarginine in Turkish pre-eclamptic women without fetal growth retardation. *J Obstet Gynaecol Res* **36**, 495-501 (2010).

25 Yaghmaei, M. et al*.* Endothelial nitric oxide synthase gene Glu298Asp polymorphism and risk of preeclampsia in South East of Iran. *Afr J Biotechnol* **10**, 10712-10717 (2011).

26 Yoshimura, T. et al. Association of the missense Glu298Asp variant of the endothelial nitric oxide synthase gene with severe preeclampsia. *J Soc Gynecol Investig* **7**, 238-241 (2000).

27 Yoshimura, T., Chowdhury, F. A., Yoshimura, M. & Okamura, H. Genetic and environmental contributions to severe preeclampsia: lack of association with the endothelial nitric oxide synthase Glu298Asp variant in a developing country. *Gynecol Obstet Invest* **56**, 10-13 (2003).

28 Yu, C. K. et al. Endothelial nitric oxide synthase gene polymorphism (Glu298Asp) and development of pre-eclampsia: a case-control study and a meta-analysis. *BMC Pregnancy Childbirth* **6**, 7 (2006).

29 Zdoukopoulos, N., Doxani, C., Messinis, I. E., Stefanidis, I. & Zintzaras, E. Polymorphisms of the endothelial nitric oxide synthase (NOS3) gene in preeclampsia: a candidate-gene association study. *BMC Pregnancy Childbirth* **11**, 89 (2011).

30 Zhang, Z. H. et al.Study on eNOS gone and MTHFR gene polymorphisms in preeclampsia. *Chinese Journal of Birth Health & Heredity* **15**, 21-23 (2007).

31 Ben Ali Gannoun, M. et al. Association of common eNOS/NOS3 polymorphisms with preeclampsia in Tunisian Arabs. *Gene* **569**, 303-307 (2015).

32 Chen, Y., Wang, D., Zhou, M., Chen, X. & Chen, J. Polymorphisms of the endothelial nitric oxide synthase gene in preeclampsia in a Han Chinese population. *Gynecol Obstet Invest* **77**, 150-155 (2014).

33 Seremak-Mrozikiewicz, A., Drews, K. & Mrozikiewicz, P. M. The -786T/C polymorphism of the endothelial nitric oxide synthase gene in preeclampsia. *Eur J Obstet Gynecol Reprod Biol* **138**, 118-119 (2008).

34 Seremak-Mrozikiewicz, A. et al. The significance of -786T > C polymorphism of endothelial NO synthase (eNOS) gene in severe preeclampsia. *J Matern Fetal Neonatal Med* **24**, 432-436 (2011).

35 Bashford, M. T., Hefler, L. A., Vertrees, T. W., Roa, B. B. & Gregg, A. R. Angiotensinogen and endothelial nitric oxide synthase gene polymorphisms among Hispanic patients with preeclampsia. *Am J Obstet Gynecol* **184**, 1345-1350; discussion 1350-1341 (2001).

36 Benedetto, C. et al*.* Synergistic effect of renin-angiotensin system and nitric oxide synthase genes polymorphisms in pre-eclampsia. *Acta Obstet Gynecol Scand* **86**, 678-682 (2007).

37 Chen, L. K. et al*.* Polymorphisms in the endothelial nitric oxide synthase gene may be protective against preeclampsia in a Chinese population. *Reprod Sci* **14**, 175-181 (2007).

38 Mozgovaia, E. V., Malysheva, O. V., Ivashchenko, T. E. & Baranov, V. S. Genetic predisposition to pre-eclampsia: Polymorphism of genes involved in regulation of endothelial functions. *Balk J Med Genet* **5**, 19-26 (2002).

39 Rahimi, Z., Aghaei, A., Rahimi, Z. & Vaisi-Raygani, A. Endothelial Nitric Oxide Synthase (eNOS) 4a/b and G894T Polymorphisms and Susceptibility to Preeclampsia. *J Reprod Fertil* **14**, 184-189 (2013).

40 Salimi, S., Naghavi, A., Mokhtari, M., Noora, M. & Yaghmaei, M. Lack of relationship between endothelial nitric oxide synthase gene 4b/a and T-786C polymorphisms with preeclampsia in southeast of Iran. *Arch Gynecol Obstet* **285**, 405-409 (2012).

41 Tempfer, C. B., Dorman, K., Deter, R. L., O'Brien, W. E. & Gregg, A. R. An endothelial nitric oxide synthase gene polymorphism is associated with preeclampsia. *Hypertens Pregnancy* **20**, 107-118 (2001).
